# BioTM Buzz (Volume 6, Issue 1)

**DOI:** 10.1002/btm2.10210

**Published:** 2021-01-08

**Authors:** Aaron C. Anselmo

**Affiliations:** ^1^ Division of Pharmacoengineering and Molecular Pharmaceutics Eshelman School of Pharmacy, University of North Carolina at Chapel Hill Chapel Hill North Carolina USA

## 1 NANOPARTICLE FOR TARGETING CARTILAGE IN OSTEOARTHRITIS

Current treatments for osteoarthritis are limited in their ability to penetrate and persist within the cartilage matrix; furthermore, existing therapies primarily focus on managing pain and modulating joint function as opposed to therapeutic treatment. As such, there is a considerable need for the development of new therapeutics and delivery systems to treat osteoarthritis by slowing, stopping, or reversing the progression of the disease. In this issue of *Bioengineering & Translational Medicine*, a team led by Professor Liangfang Zhang from the Department of NanoEngineering, Department of Chemical Engineering, and the Moores Cancer Center at the University of California San Diego describes a polymer–lipid hybrid nanoparticle that encapsulates a model drug (MK‐8722) to activate proteins that regulate the metabolism of chondrocytes in the cartilage. These nanoparticles also: (i) display peptides that bind to collagen, thereby improving targeting to cartilage, and (ii) provide controlled release of the model drug, thereby providing a sustained therapeutic effect. After characterizing this nanoparticle for size, surface properties, loading, and release and confirming that the collagen‐targeted nanoparticle enhances retention in cartilage in vivo in mice, the authors demonstrated that the collagen‐targeted nanoparticle provided maintenance of 95% of the cartilage content in an osteoarthritis model compared to 83% for the nontargeted nanoparticle and 60% for the phosphate‐buffered saline‐negative control. Overall, the collagen‐targeted nanoparticles highlight the potential of using drug delivery platforms to overcome challenges related to the targeting and retention of drugs in cartilage.

DOI: 10.1002/btm2.10187

## 2 PERSONALIZED STEM CELL THERAPY EVALUATED IN A LARGE ANIMAL MODEL

Standard treatment for glioblastoma brain tumors rely on surgical resection that is then followed by chemotherapy and radiotherapy. However, an unmet need for glioblastoma treatment exists in addressing tumor recurrence after the standard treatment for this aggressive cancer. To address this unmet need, researchers in the laboratory of Professor Shawn Hingtgen from the Division of Pharmacoengineering and Molecular Pharmaceutics at the University of North Carolina at Chapel Hill had previously developed a cell therapy platform that utilized the cancer‐homing abilities of induced neural stem cell platform to deliver tumor necrosis factor‐related apoptosis‐inducing ligand (TRAIL), a protein that can induce apoptosis in cancer cells. In this study, the authors translate this existing cell therapy platform to a large animal canine model, demonstrating key aspects of feasibility, manufacturing, and administration. Skin biopsies from canines were used to create a personalized canine induced neural stem cell therapy that was genetically engineered to deliver TRAIL and thymidine kinase. The authors demonstrate that this personalized cell therapy could be administered to canines using two clinically relevant delivery methods (within a biocompatible matrix in postsurgical cavities or through a lateral ventricle catheter). Through evaluation of cognitive function, blood chemistry, and histology, it was determined that the induced neural stem cells did not cause any toxicities. Finally, through imaging analysis using stem cells functionalized with ferumoxytol, it was postulated that the persistence of the autologous personalized stem cell therapy could last for more than 60 days in canines. Collectively, this study evaluates important factors and considerations for the clinical translation of novel cell therapies that can enable the persistent and prolonged treatment of glioblastoma.

DOI: 10.1002/btm2.10171

## 3 PRECISION NANOPARTICLES

A recent article from Professor Robert Langer of the Massachusetts Institute of Technology, Professor Nicholas Peppas of The University of Texas at Austin, and Professor Michael Mitchell of the University of Pennsylvania reviews the field of nanoparticle drug delivery. In this review, the authors discuss how the design of nanoparticles influences their interactions with various biological barriers. The authors conclude the article with an outlook that details how nanoparticles, and how engineering the nanoparticle design for specific applications, could potentially lead to unique opportunities for using nanomedicines in personalized medicine.

*Mitchell, M. J., Billingsley, M. M., Haley, R. M., Wechsler, M. E., Peppas, N. A., and Langer, R. Engineering precision nanoparticles for drug delivery. Nature Reviews Drug Discovery (2020)*; DOI: 10.1038/s41573‐020‐0090‐8

